# Do inconsistent mental models impact performance? Moderating effects of managerial interpretation and practice sets

**DOI:** 10.3389/fpsyg.2023.1110785

**Published:** 2023-04-04

**Authors:** Ying Zhao, Yuan Gao, XiLing Hao, FangFang Ren

**Affiliations:** ^1^School of Business, Zhengzhou University of Aeronautics, Zhengzhou, China; ^2^Collaboration Innovation Center of Aviation Economy Development, Zhengzhou, Henan, China; ^3^College of Business Administration, Anhui University of Finance and Economics, Bengbu, China

**Keywords:** inconsistent mental models, interpretation modes, practice sets, deviant cognition, execution and innovation performance

## Abstract

Deviant cognition, referring to team members' different understanding of goals or rules, results in inconsistent mental models among the team. Although previous studies have examined the negative effects of inconsistent mental models on deviant behavior and performance in the workplace, they have failed to consider their positive effects and moderating mechanisms, thus limiting our understanding of how to manage inconsistent mental models and deviant cognition. To address this research gap, this study builds on the interpretation and information processing theory, which regards mental models as the result of information processing, especially involving interactions where interpretation of the information is required. The study initially recruited 174 team managers as participants to identify instances of managerial interpretation. The team managers' interpretation modes were then categorized into four types (absorb, shift, limit, and explore), and a questionnaire was developed to measure them. The moderating effects of the modes on execution and innovation performance were also examined. Matched data were then collected from interviews with 104 team managers and 312 of their team members. The regression results showed that absorb, shift, and limit interpretation modes, as well as the practice sets involving managers and members, attenuated the negative relationship between inconsistent mental models and execution performance. The explore interpretation mode and the practice sets enhanced the positive relationship between inconsistent mental models and team innovation. The findings of this study help to understand the cognitive level of deviance in teams and the moderating effects of managerial interpretation on the relationship between deviant cognition, or inconsistent mental models, and performance, suggesting the need to study and utilize the positive roles of inconsistent mental models or deviance through managerial interpretation. The results also call for firms to train managers' interpretation skills and design close working links with team members.

## Introduction

To adapt to rapid changes, organizations have increasingly adopted flexible units such as teams and project groups (Guzzo and Dickson, 1996). With the impact of COVID−19, remote teams have become popular, with team members working online and in dispersed locations instead of face-to-face. Task cooperation is greatly influenced by members' inconsistent interpretation of company and team goals, which increasingly relies on a shared understanding of those goals because of the need to work autonomously when collaborating remotely (Costa et al., 2021). However, forming a shared common understanding among members, known as a team mental model (Cannon-Bowers and Sales, 2001), has become more difficult with fewer opportunities for face-to-face interaction. As it is increasingly common for individual team members to develop deviant perceptions of a team's common goals and workflows, the corresponding effects of inconsistent mental models have become important research issues (Nguyen and Huang, 2022). Further research on the impact of inconsistent mental models and the mechanisms for managing them is urgently needed. Therefore, the current study analyzed the positive and negative effects of inconsistent mental models on team performance, and how mental models can be managed to promote their positive effects and minimize their negative effects.

Inconsistent mental models are a result of team members' divergent understandings of team goals, tasks, and relationships, which can develop when members work in different contexts and locations, have diverse working hours, and experience different team cultures or supervisors (Narayanan and Moon, 2022). “Deviant cognition” is defined as a perceptional departure from organizational goals and norms, leading to inconsistent mental models of the team's goals and norms. However, while inconsistent mental models have been shown to hinder cooperation, produce destructive deviant behaviors, and lower performance (Peterson, 2002), they can also promote new ideas and other constructive behaviors that benefit organizations (Balogun, 2003; Sharma and Chillakuri, 2022). Although, previous research has mainly focused on the negative effects, neglecting the constructive or positive roles of inconsistent mental models and deviant cognition (Tekmen and Kaptangil, 2022). Furthermore, research on team mental models has focused on the effectiveness and content of shared mental models, assuming that they are formed by static factors such as demographic composition or the team's work plans and neglecting dynamic factors such as the interactive process of forming and transforming mental models (Lee et al., 2019). The transformation from inconsistent mental models to shared mental models requires interpersonal interaction and timely management. Therefore, from a theoretical perspective, there is an urgent need for research on the two-sided effects and the management or moderating mechanisms of inconsistent mental models among team members.

**Figure 1:**
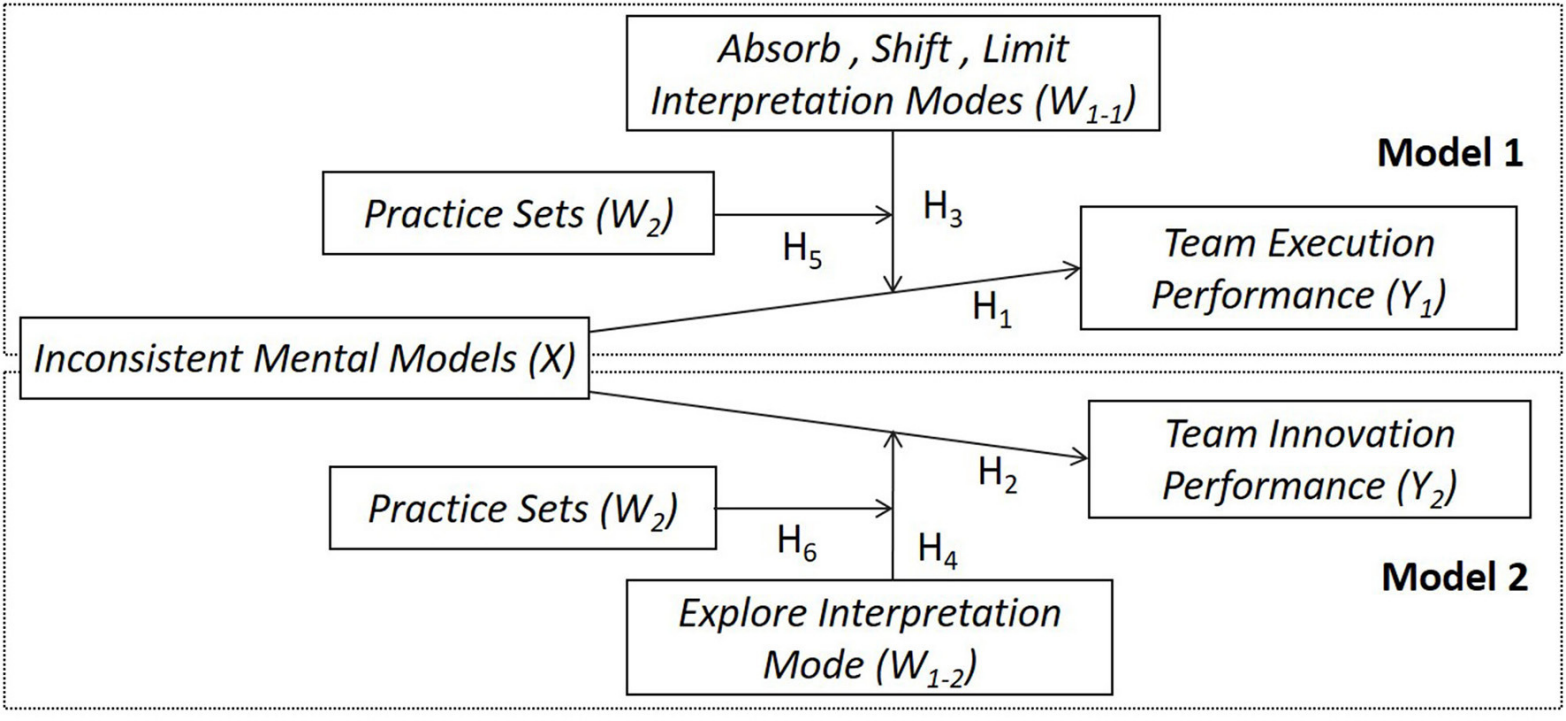
Research model.

As the core processes of cognition, interpretation, and sense-making are crucial to the construction of mental models that deviate from those of the organization, organizational support and exposure to colleagues' interpretations should reduce deviant behaviors and the negative effects of such inconsistent mental models (Weick, 1995; Tuzun et al., 2017). The purpose of the current study was to investigate the co-influence of team managers' interpretations and inconsistent mental models on team effectiveness, and the moderating effect of interpretation on the relationship between inconsistent mental models and performance in particular. To this end, we developed a questionnaire survey to gather data and determine the co-effects of interpretation and inconsistent mental models on team performance. The research model is depicted in Figure 1.

The current study focused on potential ways of decreasing the negative effects and enhancing the positive effects of inconsistent mental models and workplace deviance on organizational performance. We hope that our examination of the positive effects of inconsistent mental models on performance will alert researchers to another side of deviant cognition and behavior, and that future studies will consequently explore the moderating or managing mechanism of deviance. Furthermore, we hope our questionnaire on managers' interpretation methods might promote further leadership research and highlight the significance of specific leader behaviors, including colleagues' interpretations of the organization's goals and organizational support, especially among team managers.

From a practical standpoint, the study sheds light on the positive roles of inconsistent cognition and deviance, rather than just focusing on the negative aspects. It reminds firms to invest in developing their managers' interpretation skills and to design organizational structures that promote close working relationships between managers and team members.

## Theory and hypothesis development

### Deviance and inconsistent mental models

Workplace deviance is divided into two levels, cognitive and behavioral, with evidence of their different effects revealed over three decades of research (Puffer, 1987; Griffin et al., 1998; Fox et al., 2001; Renn et al., 2005; Sharma and Chillakuri, 2022). At the behavioral level, workplace deviance is defined as counterproductive actions, such as failing to follow instructions or doing work incorrectly, which are self-interested, unethical, and violate formal organizational rules (Griffin and Lopez, 2005; De Clercq et al., 2021). However, recent studies on the cognitive level of deviance have considered it as a constructive departure from organizational beliefs or mental models, which can lead to positive behaviors such as creative ideas and beneficial suggestions (Kibirango et al., 2017). While the negative impact of deviance at the behavioral level is widely recognized, the positive side of cognitive deviance cannot be ignored. Therefore, following Kim and Choi (2018) suggestion that research should focus more on the role of cognition in deviance and, in particular, on the two-sided effects of inconsistent mental models, our study aimed to expand the research on deviant workplace behaviors in a new, constructive direction toward deviant workplace cognition, which means inconsistent mental models in the current study.

### Inconsistent mental models and team effectiveness

Shared cognition about team goals and knowledge, represented as mental models, is crucial for team effectiveness. Mental models enable members to interpret information in similar ways and guide their cooperative behavior. Mental models have been used to explain why some teams perform better than others and are considered an explanatory and predictive factor for performance (Cannon-Bowers and Sales, 2001). However, with the increasing work autonomy in the workplace, inconsistent mental models become normal and can also lead to low performance. When members have different interpretations of goals and tasks, they naturally form deviant cognition and behaviors. Managers at different hierarchical levels have been shown to understand strategies in different ways: higher-level managers tend to consider the whole strategy, whereas middle managers tend to focus on specific tasks (Floyd and Wooldridge, 1994, 1997; Gagnon et al., 2008). The resulting inconsistent mental models can lead to reduced cooperation and execution performance (Smith-Jentsch et al., 2005; Banks and Millward, 2009). Therefore, we hypothesized that inconsistent mental models have a negative effect on team execution performance:

**Hypothesis 1:** Inconsistent mental models negatively impact team execution performance.

Although many researchers have studied the negative effects of inconsistent mental models and advocated for shared cognition in teams (Kraiger and Wenzel, 1997; Smith-Jentsch et al., 2005), some studies have highlighted both the inevitability of inconsistent mental models and their positive aspects. For instance, complex team tasks cannot be accomplished unless team members have different specialized skills and information perceptions (Banks and Millward, 2009). In a remote work environment, complex tasks require team members to think independently so that they are able to react to rapid changes and drive constant innovation. This can result in team members feeling distant and having insufficient information to develop accurate perceptions of each other, leading to inconsistent mental models that require effective communication to resolve (Handke et al., 2021). From a cognitive perspective, it is the novel interpretation of information that changes the original cognition and generates new ideas, technology, products, or services (Sharma and Chillakuri, 2022). Inconsistent mental models create a pool of novel interpretations for an organization. Van de Ven and Poole (1995) argued that persistent innovation depends on an organization's ability to effectively develop diverse mental models or cognition among its members, while Dougherty et al. (2000) found that sense-making and new interpretations of events drive change and innovation by promoting diverse cognition or inconsistent mental models. Hence, we hypothesized that inconsistent mental models have a positive effect on team innovation performance:

**Hypothesis 2:** Inconsistent mental models enhance team innovation performance.

In summary, inconsistent mental models have two-sided effects: on the one hand, they can hinder cooperation and decrease execution performance, while, on the other hand, they can enhance innovation performance.

### Interpretation and mental models

From the perspective of information processing theory, mental models are a type of cognitive schema and were defined by Cannon-Bowers and Sales (2001) as “shared cognition.” Cognitive processes typically consist of four steps, namely, stimulation, attention, interpretation, and storage. When an individual is stimulated by various types of information, they interpret the main cues according to existing schemas that help to form a new cognitive frame or mental model. Mental models simplify cognitive processing as they provide a schema that can facilitate interpretation, as the meaning is stored in the intrinsic schema, and thus speed up decision-making (Daft and Weick, 1984). Mental models stem from individuals' attention and interpretation processes, leading to inconsistent mental models. However, in the next cognitive cycle, members are influenced by organizational interpretation systems such as the organizational culture and communication with others. Thus, the effect of mental models on team performance is moderated by organizational interpretation.

The interpretation provided by team managers is at the core of organizational interpretation. Regnér (2003) highlighted the role of managers in interpretation, stating that because frontline workers are exposed to various flexible and changing needs, they receive different cues and form diverse inconsistent mental models. They need to be guided by their manager's interpretation to understand why, what, and how to proceed. The interpretation comprises two facets: strategy goals, steps, and organizational characteristics such as culture, images, and rules; and members' perceptions, which are influenced by colleagues' interpretations (Boswell, 2006; Tarakci et al., 2014). Daft and Weick (1984) proposed the following four modes that managers generally use to interpret the external environment: rational analysis, subjective setting, limited, and undisciplined. In the rational analysis mode, managers tend to use economic benefits to guide members' interpretations; in the subjective setting mode, they are guided by their beliefs and the organizational culture; in the limited mode, they use a cognitive frame; and in the undisciplined mode, they use nothing, allowing members to perceive and form their cognition by themselves. Other studies have found that managers can promote innovation and new ideas through paradoxical execution, whereby members are allowed to engage in deviant behavior or business (Brown, 2000; Lüscher and Lewis, 2008). For example, marginal products may be allocated fewer resources for development and are not included in the organization's reward system; however, if managers are open to members' deviant ideas, potential new product markets might be identified. Maitlis and Christianson (2014) studied the specific sense-making processes of interpretation, including talking and metaphor, and divided the four modes of interpretation into two categories: the rational and limited modes of interpretation guide members to maintain cognition consistent with the organization, while the subjective setting and undisciplined modes promote new cognition and innovation in the next cycle. In the current study, we developed an interpretation mode scale based on our finding of four interpretation modes, namely, absorb, shift, limit, and explore. In response to members' inconsistent cognition, managers may i) convey their rational viewpoint and ask members to execute commands and absorb formal rules into their workplace behaviors (absorb mode); ii) talk about personal benefits to shift inconsistency into self-interest (shift mode); iii) provide only an analysis framework and require members to come to a conclusion (limit mode); or iv) allow members to explore and develop their own views (explore mode). The first three interpretation modes aim to guide members to align with the organizational standpoint to improve execution performance, while the last mode aims to discover different perspectives from those of the organization to enhance innovation. In conclusion, interpretation modes moderate the effects of inconsistent mental models, as proposed in the following hypotheses:

**Hypothesis 3:** The absorb, shift, and limited interpretation modes mitigate the negative relationship between inconsistent mental models and team execution performance.

**Hypothesis 4:** The explore interpretation mode enhances the positive relationship between inconsistent mental models and team innovation performance.

### The boundary of the moderating role of interpretation modes

Based on the small world theory developed by Duncan and Watts (2011), there is a general consensus that members transfer information based on their objective relationship networks such as their living space or working group. In other words, members are more effectively influenced by their partners in actual life or work than by strangers. There is more effective information transfer in groups of members with similar careers. Similarly, based on Heidegger's habitat philosophy, Chia and Holt (2006) regarded the objective work environment as an effective place for communication, suggesting that it allows information to be effectively transmitted between members and their coworkers. Managers who work closely with members have a significant impact on their communication, as the common environment and events are important links for sharing beliefs and views.

In the field of management, in which managers are regarded as the key connecting nodes in the communication network and the relationship between top managers and team members, many researchers have studied the roles of managers' interpretation activities. Rouleau (2005) found that managers convey strategy intentions to members through their interpretation and communication skills to foster cooperation in organizations. Beck and Plowman (2009) argued that managers' interpretation activities facilitate organizations to learn and innovate, as open questions and guidance in the interpretation process inspire new ideas and solutions. The practical strategy perspective views strategy as “an organization's possession,” suggesting that effective strategy formation and execution occur through strategy-related activities, such as forums or conversations, which promote discussion and decision-making about strategy direction (Jarzabkowski et al., 2007). Furthermore, Hydle (2015) regarded common organizational activities as practice sets between managers and members: when members take part in workplace activities such as developing work plans, attending meetings, and solving problems together with their manager, they are more likely to be influenced by their manager's interpretation or views; in other words, managers can easily guide members' cognition through their interpretation of information.

Based on these previous studies, our fifth and sixth hypotheses are given as follows:

**Hypothesis 5:** The practice sets between managers and members moderate the influence of managers' interpretation on the relationship between inconsistent mental models and team execution performance.

**Hypothesis 6:** The practice sets between managers and members moderate the influence of managers' interpretation on the relationship between inconsistent mental models and team innovation performance.

Our overall research model is shown in Figure 1.

## Method

### Procedure and participants

In this study, we developed a questionnaire to measure managers' interpretation modes and then examined the moderating effects of interpretation and practice sets on the relationship between inconsistent mental models and performance. To do so, we first conducted qualitative research to collect examples of events that needed interpretation and then identified numerous specific interpreting behaviors and strategies. This was followed by exploratory factor analysis of the resulting data from 174 team managers to identify the styles and factors of interpretation events in each team. Next, we tested the model shown in Figure 1 using quantitative research. We randomly selected 104 team managers and 312 corresponding team members (3 members per team). Our research focused mainly on sales teams in the food industry, where team managers and members often work remotely and communicate through electronic tools such as WeChat and email, forming virtual teams. These teams face a constantly changing work context and various inconsistent mental models.

### Development of the managers' interpretation questionnaire

To gather substantial interpretation content, we limited our qualitative research interviewees to managers in the food industry. To identify which manager interpretation behaviors effectively reduce inconsistency and promote performance, we asked top human resource managers and other functional departments to recommend excellent managers in sales and marketing teams based on their performance over the last 3 years. We chose 22 excellent sales team managers in the food industry and asked them to share ambiguous events where they needed to explain the characteristics of those events to their team members and identify the effective interpretation methods they used. After summarizing the events and characteristics, we finally acquired 24 events that were often interpreted and identified more than 10 interpretation modes. These events were either relevant to organizational institutions or members' interests. When dealing with formal and clear rules, and team members' direct interests were involved, managers usually guided team members to accept the rules. If members had contrary opinions about ambiguous rules that concerned indirect benefits, managers sometimes provided a broad framework, such as the organizational culture or mission, or allowed members to consider and explain the rules themselves. The two factors that influenced interpretation modes were the degree of clarity of the rules involved and whether they were relevant to the members' direct interests.

Next, to develop the questionnaire, we used a snowball method to ask the 22 managers to recommend another 59 team managers to participate in our survey. We received responses from 81 managers in total, 67 of whom were men and 14 were women, with an average age of 32.7 years; 56 of the managers worked in business sections and 25 in function sections. Our survey consisted of the following two main questions: what kinds of events did they often need to explain to members and how did they interpret or explain those events to their team members?

Initially, we randomly chose 16 of the 81 managers to discuss their understanding of the meaning of interpretation, which differs from the meaning in daily life, and when the meaning was ambiguous, we modified the term in the survey. We then sent the modified questionnaire outline to the participants. The outline focused on ambiguous events that were not understood by members or had various interpretations. We asked managers to answer how they would interpret or guide members to overcome their differences in cognition and to provide examples.

The interviews were conducted over 2 months (January to February 2019), and from the data, we obtained 69 events and 221 interpretation modes. A doctoral candidate and a marketing manager then separately (back-to-back) merged the events and modes into 20 categories comprising 52 interpretation modes and 18 categories comprising 61 interpretation modes. We then discussed their different classifications and combined them into 16 categories (40 interpretation modes) based on abstraction and universality principles. The categories were then evaluated by three other doctoral candidates, and the level of agreement among raters was calculated using the RWG coefficient, which was 0.89. Finally, we used the events and 40 interpretation behaviors to create a pilot questionnaire, which was used to collect data for exploratory factor analysis (EFA). We asked the participants to rate their agreement with each item on a 5-point Likert scale, ranging from “completely agree” (5) to “completely disagree” (1).

To implement the EFA, we expanded the number of participants to 174 randomly selected, excellent team managers who were recommended by other HR departments. The data were collected over 3 months and the questionnaire was completed either in the field or by email or WeChat. To analyze the data, we first calculated the item–total correlations between the items and the total scores. Based on the norms suggested by Jean and Reynolds (1980), we removed 11 items that had item–total correlations lower than 0.35, leaving 29 items. Then, to account for possible relationships between the participants' answers, we performed the Kaiser–Meyer–Olkin (KMO) test with oblique rotation. The results indicated that it was appropriate to use EFA: KMO value = 0.677, χ^2^ = 3,021.05, *df* = 406, *p* < 0.0001. Based on the rule of having more than one eigenvalue and assessment of the scree plot and the factor loading, we extracted four factors. After limiting the factors to four and the factor loading to >0.4, we found that the four factors explained 52.1% of the total variance. After deleting 5 items with a factor loading coefficient of <0.4, removing 8 reverse items and 4 repetitive items, we were left with 12 items measuring “managers' interpretation modes.” The items and their factor loading coefficients are shown in Table 1.

**Table 1:** Managers' interpretation mode questionnaire.

**Interpretation mode**	**Item**	**Loading coefficient**
Limit	I often tell him that this embodies the excellent culture in our firm.	0.777
	I often review top managers' public service and spirit with them.	0.753
	I often show him the great images and culture of our firm in society.	0.727
Absorb	I provide him with help and explanations about business.	0.790
	I care about his life, recognizing his work problems through the events.	0.776
	I usually allow members to help each other in business, avoiding poor performance.	0.600
Shift	I explain to him that matching of position power and duty benefits his development.	0.711
	I often tell members that obeying rules helps communication between superior and subordinate, which decreases their risk in decision making.	0.651
	I emphasize the openness and fairness of institutions.	0.607
Explore	I discuss new work assignments with team members.	0.786
	I reveal business data to team members, discussing the market outlook with them.	0.717
	I let members know that I might suggest to a superior that we need to improve cooperation to ensure a smooth work flow.	0.652

Based on our interviews and the results of the EFA, combined with the research of Daft and Weick (1984), we identified the four interpretation modes, namely, absorb, shift, limit, and explore. The absorb mode indicates that when members face conflicts with specific formal institutions that directly influence members' benefits, such as the organization's performance assessment system, team members should be guided to accept the organizational rules. The shift mode suggests that when facing conflicts with formal institutions and indirect benefits, such as unconditional returns or other free services, managers should encourage team members to consider the benefits of the rule (e.g., attracting customers). The limit mode means that when facing conflicts with informal institutions that are indirectly beneficial, such as public welfare donation, members should be guided to discuss related events and use the institution's culture or mission to guide their understanding. The explore mode means that when facing conflicts with informal institutions that directly impact members' interests, such as business reengineering or new product development, managers should not guide members' thoughts, but rather explore their ideas and openly discuss them to find better solutions.

### Measures

#### Interpretation modes

We randomly selected 120 team managers to complete the managers' interpretation questionnaire outlined in Table 1. All participants were asked to report their actual behaviors using a 5-point scale. The responses were divided into two groups: one included the absorb, shift, and limit modes, which are intended to form consistent cognition to improve team execution performance, and the other included only the explore mode, which is beneficial for team innovation.

#### Inconsistent mental models

The mental models included both task work and teamwork aspects. Task work focuses on team members' cognition related to their assigned behaviors and tasks (Mathieu et al., 2000; Smith-Jentsch et al., 2005), whereas teamwork involves communication, relationships, and coordination within teams (Ilgen, 1999). The survey was completed by 360 members from 120 teams; after removing incomplete surveys and those with consistent answers, the data of 312 members from 104 teams were available for analysis.

First, we measured the extent to which members' mental models about task work were inconsistent, which was related to members' approaches for coping with tasks. We interviewed marketing and sales managers and identified three widespread events or tasks and their associated coping approaches (listed in detail in Appendix A), i.e., Event 1: poor sales of new products, associated with nine coping approaches; Event 2: quality complaints from customers, associated with six coping approaches; Event 3: doubts about product quality and the brand, associated with six coping approaches. Then, we asked the team members to rank how important they thought each coping approach was for solving the event specific to their team task based on the effect of the approach on innovation and new product sales. The approaches were scored by their order of importance, with the most important approach receiving the maximum score (as stated in Appendix A).

Next, we used the squared Euclidean space method (Webber et al., 2000) to index the degree of inconsistency between members' mental models by calculating the difference in rank orders. The calculation steps were given as follows:


$$\begin{array}{r} SD_{12}=\sqrt{\sum_{j=1}^{n}{\left(a_{1j}-a_{2j}\right)}^{2}},\;index\;of\;Euclidean\;distance\;between\\ member\;1\;and\;member\;2\;(1.1); \end{array}$$

$$\begin{array}{r} SD_{23}=\sqrt{\sum_{j=1}^{n}{\left(a_{2j}-a_{3j}\right)}^{2}},\;index\;of\;Euclidean\;distance\;between\\ member\;2\;and\;member\;3\;(1.2); \end{array}$$

$$\begin{array}{r} SD_{13}=\sqrt{\sum_{j=1}^{n}{\left(a_{1j}-a_{3j}\right)}^{2}},\;index\;of\;Euclidean\;distance\;between\;\\ member\;1\;and\;member\;3\;(1.3); \end{array}$$

$$\begin{array}{r} SD_{ti}=\frac{SD_{12}+SD_{23}+SD_{13}}{3},\;index\;of\;total\;Euclidean\\ distance\;in\;a\;team\;and\;index\;of\;inconsistent\;mental\;models\;about\\ task\;work\;in\;a\;team\;(1.4); \end{array}$$

where *j* refers to each coping approach for every event (*j* = 1…9 in event 1, *j* = 1…6 in events 2 and 3); *t*_*i*_ refers to a team i; and *n* refers to the number of coping approaches for each event (*n* = 9 for event 1, *n* = 6 for events 2 and 3).

Second, we measured the extent to which members' mental models about teamwork were inconsistent. This measure was related to members' cognition about relationships and cooperation within the team. Mathieu et al. (2000) asked team members to indicate the number of workflows they focused on to measure the level of interaction and teamwork cognition, indexed by the correlations between the interviewees' assessments and the team's characteristics (such as active cooperation and communication). However, Smith-Jentsch et al. (2005) argued that this method was somewhat abstract and lacked specificity, which could lead to unreliable answers. To address this issue, Smith-Jentsch et al. (2005) developed a goal-related method to assess the teamwork mental model, which more effectively estimated the interactions within a team by asking members to rank the influence of others' behaviors on themselves.

Based on Smith-Jentsch et al. (2005) research, by asking more than 20 team managers and members, we first identified the four actions of others that mostly influence one's new product markets and impact one's sales, which are shown in Appendix B. The scores could be positive or negative, with positive values indicating that members felt they helped each other and negative values indicating that they felt they hindered each other. However, regardless of whether the values were positive or negative, the scores indicated that team members were deeply interdependent and felt like a family. To quantify this, we calculated the average of the absolute value of the scores given by three team members and used the reciprocal of the average absolute value as the inconsistent mental model of teamwork. The computational formula is given as follows:


$$\begin{array}{r} GS_{t}=\frac{\sum_{j=1}^{4}\sum_{i=1}^{3}\left|a_{ij}\right|}{12}\;index\;of\;shared\;feeling\;of\\ interdependence\;(1.5); \end{array}$$

$$\begin{array}{r} MH_{t}=\frac{1}{GS_{t}}\;index\;of\;the\;inconsistent\;feelings\;of\\ interdependence\;(1.6); \end{array}$$

Where *i* denotes the members in the team (*i* = 1,2,3); *j* denotes each item (*j* = 1,2,3,4); *a*_*ij*_ denotes the *j*^th^ member's assessment value in the i^th^ department; and *t* denotes each team.

Finally, we calculated the total number of inconsistent mental models (*M*) by summing *SD*_*ti*_ (from Formula 1.4) and *MH*_*t*_ (from Formula 1.6) above. The specific formula is given as follows:


$$M=SD_{\text{ti}}+MH_{\text{t}}\ldots \ldots \ldots \ldots \ldots \ldots \ldots \ldots \ldots \ldots \ldots \ldots \;(1.7)$$

#### Execution performance of the team

The team's execution performance was measured by the number of fixed tasks that the team completed, assessed by the superior manager based on the average performance over the last 6 months. The scores were divided into the following four grades: excellent (exceeded expectations and assigned a value of 4), fine (slightly exceeded expectations), qualified (met expectations), and unqualified (did not meet expectations and assigned values of 1–4).

#### Innovation performance of the team

Because different firms use different methods to evaluate innovation, it was difficult to gather objective innovation performance data. Instead, we used a subjective assessment whereby team members reported their own creativity. We used the team innovation questionnaire by Shin et al. (2017) and Jial et al. (2014), which included questions such as “Have you ever suggested different product or service ideas?”, “Is the current product or service idea outdated compared with your suggestions?”, and “Have you ever suggested ideas that would improve the current product or service?” Three members of each team answered these questions, and the average value was used as the index of team innovation performance.

#### Practice sets of the team

Based on the study of Hydle (2015), we measured practice sets using the number of common activities in which both a team member and the team manager participated, asking them the following question: “How many times do you resolve work issues with your team manager each month?” We then summed the responses of all members of each team to measure this variable.

#### Control variables

We controlled for the average age and average years of work experience of the three members of each team. Male team members were assigned a value of one, while female team members were assigned a value of zero.

## Results

The results are divided into four sections. The first section describes the sample and data collection process, the second section presents the calculation of the interpretation modes index, the third section shows the results of the test of the hypotheses in Model 1 (H_1_, H_3_, and H_5_ in Figure 1), and the fourth section presents the results of the test of our hypotheses in Model 2 (H_2_, H_4_, and H_6_ in Figure 1).

### Sample analysis

Data were gathered from 120 team managers in 3 firms over a 3-month period (May to July 2021). Three team members and their corresponding managers were interviewed from each team, resulting in 360 members participating in the survey. After eliminating invalid samples (such as incomplete or similar answers), 104 matched and valid samples remained, comprising 104 team managers and 312 team members. Of the 104 managers, 67.3% were men and 32.7% were women, with an average age of 39.4 years and an average of 11.33 years of work experience. The analysis was conducted at the team level, thus, the final sample size was 104.

### Reliability and validity of the interpretation modes scale

To assess the reliability of the interpretation modes scale, we used SPSS to calculate the alpha coefficient. The results showed that absorb, shift, limit, and explore modes had good reliability, with alpha coefficients of 0.82, 0.765, 0.672, and 0.88, respectively, all of which were >0.6, indicating sufficient reliability.

We first conducted a factor analysis to assess the convergent validity of the scale. When the 12 items were loaded onto a single factor, the loading coefficients all exceeded 0.5, indicating excellent convergent validity. Next, we used MPLUS to assess the discriminant validity of the one-, two-, three-, and four-factor CFAs. The results showed that the CFA with four factors had the best discriminant validity (Table 2). Therefore, it was appropriate to divide the managers' interpretation modes into four types.

**Table 2:** CFA of the interpretation modes scale (*N* = 104).

**Mode**		**Df**	**CFI**	**TLI**	**SRMR**
One factor	395.45	54	0.308	0.154	0.171
Two factors	320.2	53	0.458	0.325	0.160
Three factors	200.77	51	0.783	0.665	0.112
Four factors	115.03	48	0.945	0.904	0.009

Table 2 shows that the comparative fit index (CFI) and Tucker–Lewis index (TLI) values increased gradually from 0.308 and 0.154 to 0.945 and 0.904, respectively, while the standardized root mean squared residual (SRMR) value decreased gradually from 0.171 to 0.009. This confirmed that dividing the interpretation modes into four factors was the best approach.

The other variables (inconsistent mental models, practice sets, execution, and innovation performance) were measured by a collective grade calculated using the above formulas (1.1–1.7) and the gross score of all members' assessments, thus, we did not analyze their reliability and validity.

### Substantive relationships in Model 1

Model 1 (Figure 1) tested the extent to which absorb, shift, and limit interpretation modes of team managers moderated the relationship between inconsistent mental models and execution performance. Table 3 shows the descriptive statistics and correlations between all variables in Model 1. It reveals a significant negative relationship between execution performance (Y_1_) and inconsistent mental models (X, *r* = −0.182, *p* < 0.05) and a strong positive relationship between execution performance (Y_1_) and the absorb, shift, and limit interpretation modes of managers (W_1−1_, *r* = 0.529, *p* < 0.01) and practice sets (W_2_, *r* = 0.561, *p* < 0.01). These positive and negative correlations provided preliminary confirmation of the hypothesis in Model 1.

**Table 3:** Correlations and descriptive statistics of all variables in Model 1 (*N* = 104).

**Variables**	**M**	**SD**	**1**	**2**	**3**	**4**	**5**	**6**
1. Age	32.23	4.15						
2. Working years	5.88	3.03	0.693^**^					
3. Gender	0.87	0.22	0.017	0.510				
4. Inconsistent mental model (x)	7.45	2.59	−0.334^**^	−0.244^*^	0.024			
5. Interpretation modes (w_1−1_, absorb, shift, limit)	3.34	0.32	0.095	0.092	−0.053	0.027^*^		
6. Practice sets (w_2_)	6.13	2.09	−0.130	−0.060	0.165	0.207^*^	0.406^**^	
7. Execution performance (y_1_)	5.23	1.87	0.071	0.098	−0.065	−0.182^*^	0.529^**^	0.561^**^

** *p* < 0.01 and

* *p* < 0.05.

We used hierarchical multiple regression to test our study hypothesis in Model 1. Following the guidelines suggested by Hwang and Grant (2011), we first centered the interaction terms to avoid multicollinearity. The results showed that the variance inflation factor (VIF) values were between 1 and 5 and the Durbin–Watson (DW) value was close to 2, indicating that there were no issues with multicollinearity. Hierarchical multiple regression was then performed using SPSS, and the results are presented in Table 4.

**Table 4:** Hierarchical regression of execution performance on covariates, inconsistent mental models, interpretation modes (absorb, shift, and limit), and practice sets (*N* = 104).

**Predictors**	**M_0_**	**M_1_**	**M_2_**
**Covariates**			
1. Age	0.003	0.040	0.087
2. Working years	0.100	0.053	0.064
3. Gender	−0.070	−0.039	−0.095
**Independent variable**			
4. Inconsistent mental models (x)		−0.196^**^	0.156^*^
**Moderating variables and interaction terms**			
5. Interpretation modes (absorb, shift, limit, w_1−1_)		0.624^*^	0.649^***^
6. Inconsistent mental models^*^Interpretation modes (x^*^w_1−1_)		0.137^**^	0.111^*^
7. Practice sets (w_2_)			0.272^***^
8. Inconsistent mental models^*^practice sets (x^*^w_2_)			0.016^*^
9. Interpretation models*practice sets (${w_{1-1}}^{*}$w_2_)			0.150^*^
10. Inconsistent mental models^*^ interpretation modes * Practice sets (x${}^{*}{w_{1-1}}^{*}$w_2_)			0.051**
*R* ^2^	0.015	0.583^***^	0.643^***^
Δ*R*^2^	—-	0.569^***^	0.060^***^

*** *p* < 0.001,

** *p* < 0.01,

* *p* < 0.05.

First, we discuss the main effect of inconsistent mental models on execution performance. M_1_ in Table 4 shows a significant negative influence of inconsistent mental models on execution performance (β = −0.196, *p* < 0.01), thus verifying Hypothesis 1.

Second, M_1_ also verified the moderating effect of the three interpretation modes (absorb, shift, and limit) on the relationship between inconsistent mental models and execution performance. The main effect was highly positive (β = 0.624, *p* < 0.05), and the common effect of the interpretation modes and inconsistent mental models on execution performance was positive and significant (β = 0.137, *p* < 0.01), verifying Hypothesis 3. This confirmed that the absorb, shift, and limit interpretation modes alleviated the negative relationship between inconsistent mental models and team execution performance.

Third, the common moderating effects of team managers' interpretation modes (absorb, shift, and limit) and practice sets on execution performance were tested in M_2_, and the results are shown in Table 4. The interaction term between interpretation modes and practice sets had a significant effect on execution performance (β = 0.15, *p* < 0.05), indicating that they had a common influence on the outcome. In addition, the interaction term formed by inconsistent mental models, interpretation modes, and practice sets had a positive effect on the outcome variable (β = 0.051, *p* < 0.01), suggesting that together, they influenced execution performance, changing the previous relationship between inconsistent mental models and execution performance.

These results imply that when team managers have sufficient common practice with their team members, form close relationships, and apply corresponding interpretation modes (absorb, shift, and limit), they can reduce the negative effects of inconsistency, thus enhancing cooperation and execution performance. In this way, team managers can alleviate or moderate the negative impact of inconsistent mental models.

Finally, we analyzed the specific moderating effects of the absorb, shift, and limit interpretation modes and practice sets. First, we regressed execution performance (Y_1_) on inconsistent mental models (X), interpretation modes (W_1−1_), and practice sets (W_2_), and obtained the following model:


$$\begin{array}{r} Y_{1}={\left(0.101+0.096^{*}w_{1-1}-0.028^{*}W_{2}+0.025^{*}{W_{1-1}}^{*}W_{2}\right)}^{*}X+\\ 0.681^{*}W_{1-1}+0.247^{*}W_{2}+0.142^{*}{W_{1-1}}^{*}W_{2}. \end{array}$$

**Figure 2:**
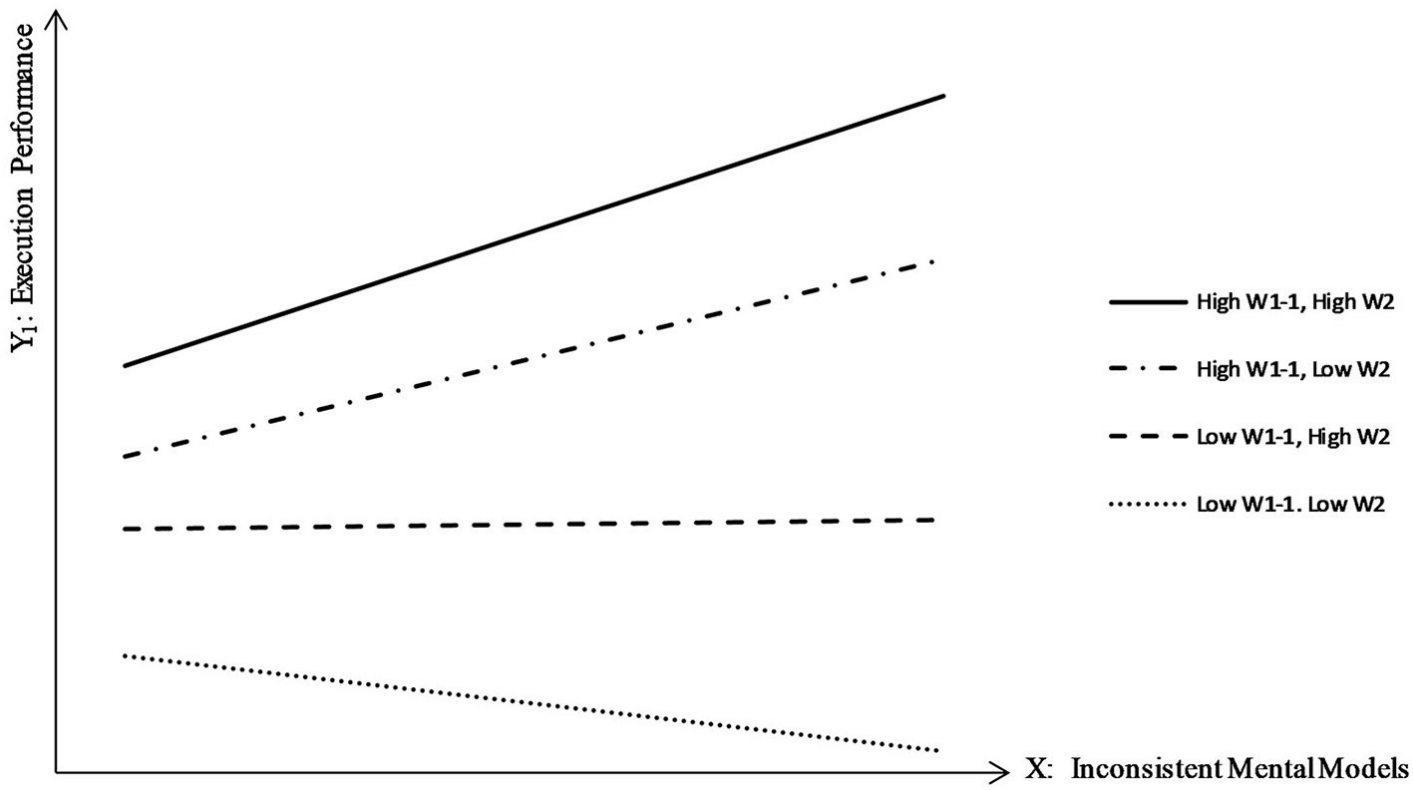
Interaction effects of interpretation modes (absorb, shift, limit)*practice sets on the relationship between inconsistent mental models and execution performance.

Then, we defined the following four types: high W_1−1_ and high W_2_, high W_1−1_ and low W_2_, low W_1−1_ and high W_2_, and low W_1−1_ and low W_2_ (illustrated in Figure 2). Their corresponding slopes are shown in Table 5, illustrating the common moderating effects of W_1−1_ and W_2_.

**Table 5:** Moderating effects of interpretation modes (absorb, shift, and limit) and practice sets.

**Types**	**Slope**
(High W_1−1_, High W_2_)	0.530
(High W_1−1_, Low W_2_)	0.389
(Low W_1−1_, High W_2_)	0.017
(Low W_1−1_. Low W_2_)	−0.187

As shown in Table 5, the coefficient of inconsistent mental models (X) on execution performance (Y_1_) was greatest and changed to a positive value when both interpretation modes (W_1−1_) and practice sets (W_2_) were high. This means that execution performance was not reduced by inconsistent mental models (X) with high W_1−1_ and high W_2_. However, for both low interpretation modes (W_1−1_) and low practice sets (W_2_), the coefficient of inconsistent mental models (X) on execution performance (Y_1_) was negative, indicating that execution performance was reduced by inconsistent mental models (X).

### Substantive relations in Model 2

In Model 2 (Figure 1), the aim was to test the extent to which the explore interpretation mode moderated the relationship between inconsistent mental models and innovation performance. We assumed that the explore interpretation mode would strengthen the influence of inconsistent mental models on innovation performance because, in this mode, managers can help members to analyze their cognition and generate new ideas, thus enhancing the positive effect of inconsistency. Table 6 shows the descriptive statistics and correlations between all of the variables in Model 2, revealing a significant positive relationship between innovation performance (Y_2_) and inconsistent mental models (X, *r* = 0.144, *p* < 0.05), and strong positive relationships between innovation performance (Y_2_) and the explore interpretation mode (W_1−2_, *r* = 0.434, *p* < 0.01) and practice sets (W_2_, *r* = 0.363, *p* < 0.01). These correlations verify the hypothesis in Model 2.

**Table 6:** Correlations and descriptive statistics of all variables in Model 2 (*N* = 104).

**Variables**	**M**	**SD**	**1**	**2**	**3**	**4**	**5**	**6**
1. Age	32.23	4.15						
2. Working years	5.88	3.03	0.693^**^					
3. Gender	0.87	0.22	0.017	0.510				
4. Inconsistent mental model (x)	7.45	2.59	−0.334^**^	−0.244^*^	0.024			
5. Explore interpretation mode (w_1−2_)	3.66	0.47	0.130	0.072	0.110	0.242^**^		
6. Practice sets (w_2_)	6.13	2.09	−0.130	−0.060	0.165	0.207^*^	0.226^**^	
7. Innovation performance (y_2_)	7.13	0.996	0.105	0.083	0.127	0.144^*^	0.434^**^	0.363^**^

** *p* < 0.01 and

* *p* < 0.05.

We used hierarchical multiple regression to further test the hypothesis in Model 2. To avoid multicollinearity, we used the centering process for interaction terms and found that the VIF values were between 1 and 4 and the DW value was close to 2, indicating no collinearity issues. The results of the hierarchical multiple regression are presented in Table 7.

**Table 7:** Hierarchical regression of innovation performance on covariates, inconsistent mental models, explore interpretation mode, and practice sets (*N* = 104).

**Predictors**	**M_0_**	**M_1_**	**M_2_**
**Covariates**			
1. Age	0.096	0.130	0.144
2. Working years	0.010	0.026	−0.016
3. Gender	0.125	0.114	0.118
**Independent variable**			
4. Inconsistent mental models (x)		0.094^*^	0.088^*^
**Moderating variables and interaction terms**			
5. Explore interpretation mode (w_1−2_)		0.165^**^	0.171^**^
6. Inconsistent mental models^*^Interpretation modes (x^*^w_1−2_)		0.046^**^	0.038^*^
7. Practice sets (w_2_)			0.363^***^
8. Inconsistent mental models^*^practice sets (x^*^w_2_)			0.027^*^
9. Interpretation modes^*^practice sets (*w*_1−2_^*^w_2_)			0.104^*^
10. Inconsistent mental models^*^interpretation mode^*^practice sets (x^*^w_1−2_^*^w_2_)			0.296^**^
*R* ^2^	0.061	0.192^***^	0.274^**^
Δ*R*^2^	—-	0.131^***^	0.082^**^

*** *p* < 0.001,

** *p* < 0.01,

* *p* < 0.05.

First, the main effect of inconsistent mental models on innovation performance was significant and positive (β = 0.094, *p* < 0.05), as shown in M_1_ of Table 7, which supports Hypothesis 2.

Second, the moderating effect of the explore interpretation mode on the relationship between inconsistent mental models and innovation performance was also confirmed. The single effect of the explore interpretation mode was positive and significant (β = 0.165, *p* < 0.01). The combined effect of the explore interpretation mode and inconsistent mental models on innovation performance was also positive and significant (β = 0.046, *p* < 0.01), which supports Hypothesis 4. This suggests that the explore interpretation mode can enhance the positive relationship between inconsistent mental models and innovation performance.

Third, the combined moderating effects of the explore interpretation mode and practice sets on innovation performance were tested, and the results are shown in M_2_ of Table 7. The interaction term of the explore interpretation mode and practice sets had a significant effect on innovation performance (β = 0.104, *p* < 0.05), indicating their joint influence on innovation. Moreover, the interaction term formed by inconsistent mental models, the explore interpretation mode, and practice sets had a positive effect on innovation performance (β = 0.296, *p* < 0.01). This indicates that all three factors together can positively influence innovation performance.

Finally, we examined the specific moderating effects of the explore interpretation mode and practice sets. The regression of innovation performance (Y_2_) on inconsistent mental models (X), explore interpretation mode (W_1−2_), and practice sets (W_2_) resulted in the following relationship:


$$\begin{array}{r} Y_{2}={\left(0.041+0.019^{*}W_{1-2}-0.181^{*}W_{2}+0.075^{*}{W_{1-2}}^{*}W_{2}\right)}^{*}X-\\ 0.197^{*}W_{1-2}+0.073^{*}W_{2}+0.154^{*}{W_{1-2}}^{*}W_{2.} \end{array}$$

**Figure 3:**
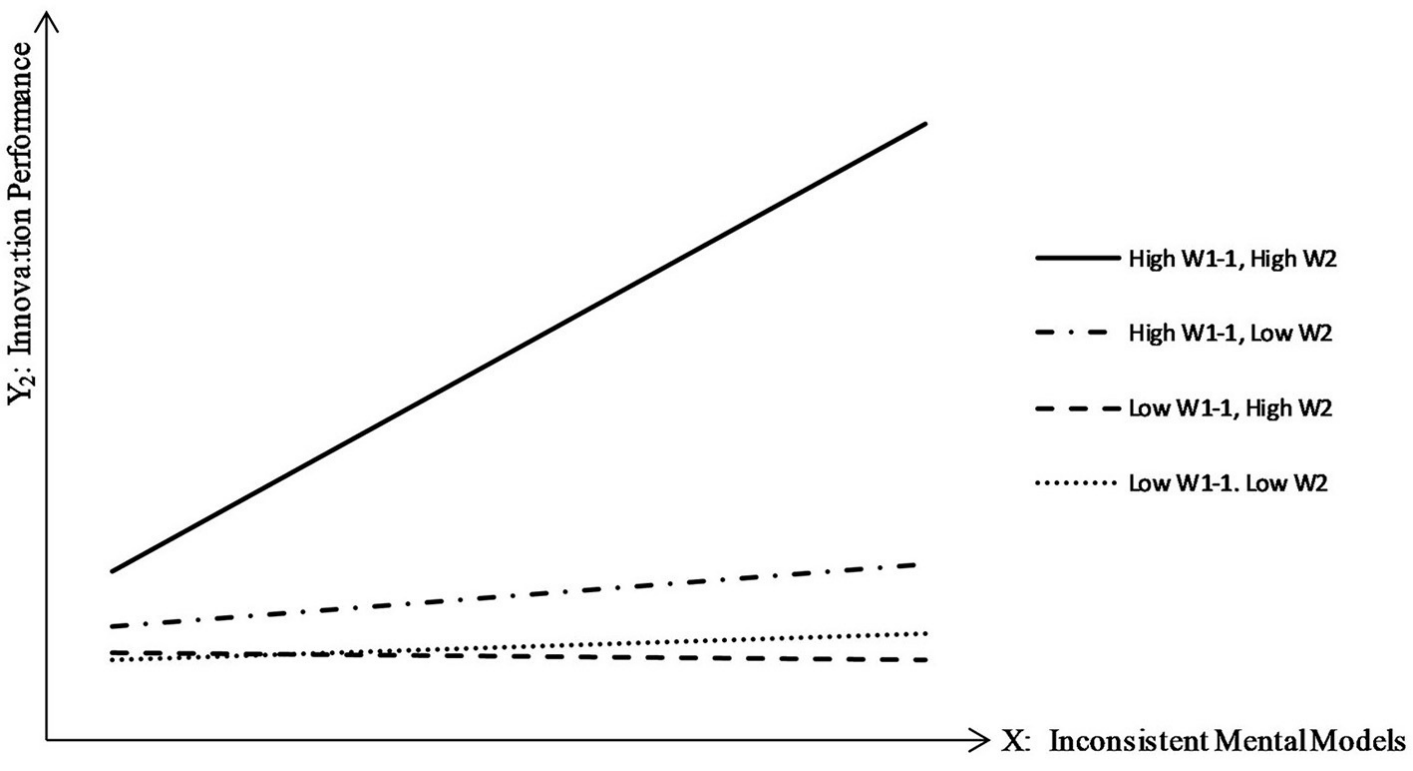
Interaction effects of the explore interpretation mode*practice sets on the relationship between inconsistent mental models and innovation performance.

We divided the results into four types (high W_1−2_ and high W_2_, high W_1−2_ and low W_2_, low W_1−2_ and high W_2_, and low W_1−2_ and low W_2_), and the corresponding slopes are shown in Table 8. The four types are also depicted in Figure 3.

**Table 8:** Moderating effects of the explore interpretation mode and practice sets.

**Types**	**Slope**
(High W_1−2_, High W_2_)	0.718
(High W_1−2_, Low W_2_)	0.098
(Low W_1−2_, High W_2_)	−0.013
(Low W_1−2_. Low W_2_)	0.041

As shown in Table 8, the influence of inconsistent mental models (X) on innovation performance (Y_2_) was strongest when both the interpretation mode (W_1−2_) and practice sets (W_2_) were high, indicating the closest relationship between X and Y, and the greatest enhancement of innovation. Contrary to the results in Figure 2 and Table 5, when practice sets (W_2_) was high and the explore interpretation mode (W_1−2_) was low, inconsistent mental models (X) reduced innovation performance (Y_2_). This indicates that practice sets alone cannot improve innovation and must involve the explore interpretation mode. Indeed, without the appropriate explore interpretation mode, the close working relationships between team members only produce repetitive and underdeveloped information, which cannot benefit innovation.

## Discussion

Previous studies have often focused on analyzing the negative effects of inconsistent cognition (Smith-Jentsch et al., 2005) but have also found positive effects on innovation (Beck and Plowman, 2009); their contradictory conclusions about the impact of inconsistent cognition arose because they neglected the moderating mechanism. In this study, we argue that, with appropriate interpretation modes, inconsistent mental models or cognition can play a more positive role.

We found that different interpretation modes have varying moderating effects, suggesting that inconsistent mental models or deviant cognition can have both positive and negative effects on performance. This discovery challenges the conventional view that deviant cognition and behavior have only negative effects and opens the door to exploring the positive roles of deviant cognition in enhancing the levels of cognition. This supports Sharma and Chillakuri's (2022) finding that deviance can have positive effects on cognition. Based on information processing theory, we argued that team managers' interpretation influenced members' cognition and then verified that the absorb, shift, and limit interpretation modes were aimed at incorporating information into existing cognitive schemas to reduce the impact of diverse cognition and improve execution performance (β = 0.137, *p* < 0.01, Table 4). In contrast, the explore interpretation mode is aimed at generating new schemas to enhance the positive effects of diverse cognition on innovation (β = 0.046, *p* < 0.01, Table 7). The moderating effects were further strengthened by practice sets (β = 0.051, *p* < 0.01, Table 4; β = 0.296, *p* < 0.01, Table 7).

As hypothesized, we confirmed the significant impact of inconsistent mental models, interpretation modes, and practice sets on team performance. We found that specific interpretation modes had a significant influence on the relationship between inconsistency and outcomes. This finding confirms those of previous studies that have reported the two sides of inconsistent cognition (Dunbar and Garud, 2009; Han, 2010; Balogun et al., 2014; Maitlis and Christianson, 2014) and expands on them by focusing on the moderating or managing mechanism, which may shift the direction of future research.

Moreover, previous studies on research managers' interpretation behavior have concluded that managers use various information interpretation styles, such as fragmented and restricted sense-making (Daft and Weick, 1984; Maitlis, 2005; Yulan, 2010; Balogun et al., 2014). However, these studies lacked a scale for measuring interpretation based on actual events and the team manager context. In this study, we developed a questionnaire to assess team managers' interpretation modes based on their actual work practices. Through interviews with several managers, a pilot questionnaire, a final questionnaire, and EFA, we developed a scale with four interpretation modes, namely, absorb, shift, limit, and explore. Each mode was assessed by three items, as described in Table 1. After further examination using a different sample, as shown in Table 2, our scale showed good reliability and validity. Our study on managers' interpretation behavior adds to the literature on the organizational interpretation process by identifying specific interpretation behaviors based on the work context and emphasizing the role of interaction and collaboration in forming shared cognition. Therefore, this study expands the interpretation theory and also contributes to leadership theory by paying attention to the content of leadership rather than just the leadership style.

### Theoretical and managerial implications

This study has several theoretical and managerial implications. First, it contributes to the research on deviant behavior by highlighting the importance of the cognitive level of deviance, instead of focusing solely on deviant behaviors. Second, it sheds light on the positive side of inconsistent mental models and deviance, which were previously considered as having only negative effects. Third, our findings suggest that the management of inconsistent cognition and deviance should focus on organizational methods, such as work relationships and communication, to enhance the positive side of inconsistent mental models. Future research should explore the factors and processes that influence inconsistent mental models and deviant cognition, including mediating and moderating mechanisms, to develop a comprehensive study mode and deepen the theory. Finally, the scale of managers' interpretation modes can be useful in communication and leadership fields, as well as other studies that measure manager behaviors or communication behaviors.

### Practical implications

Our results have practical implications for managers and organizations. First, managers should be aware of their interpretation skills and choose appropriate behaviors based on the current situation. For example, when faced with clear rules, or indirect benefits with unclear rules, members should be guided to absorb and understand the information. Conversely, when dealing with unclear rules but with direct benefits, members should be encouraged to identify their own views through a collaborative exploration process. Second, the results suggest that managers should work closely with their team members to guide their cognition through interpretation. Third, organizations should build members' close working links when designing organizational structure and workflows, and this would improve communication and take use of members' diverse cognition.

### Limitations and future directions

The current study has some limitations. First, to ensure a diverse sample and a comparable level of cognition content in a similar firm context, our survey was conducted in three firms within the same food and drink industry, and the chosen managers were primarily from the marketing or sales departments, with few from functional departments. This method of sampling may have affected the generalizability of the results and should be improved in future studies by including a wider range of industries. Second, there may be other combinations of the four interpretation modes, and our study only analyzed two outcomes, which may not align with Maitlis (2005), Maitlis and Christianson (2014) theoretical research.

Future studies should aim to expand the range of participants beyond the food industry to improve the applicability of the interpretation questionnaire and the effectiveness of the moderating mechanism. In addition, participants from other positions besides sales should be included as long as they have diverse cognition, such as virtual online teams in the current digital age, to further enhance the study's generalizability. Finally, future studies should focus on potential moderating mechanisms such as the communication content, channel, or mode, which might have a strong influence on the outcome and the effects of inconsistency.

## Conclusion

The current study developed a scale of managers' interpretation modes, consisting of 12 items and 4 factors or types. The absorb, shift, and limit interpretation modes guide members to support the organization's strategy, tasks, and institutions, thereby improving cooperation and execution performance. The explore mode helps team members to generate new ideas and solutions and thus improve team innovation, but only if there is a close working relationship or a common task between the manager and the team members. The results suggest that team managers should adopt appropriate interpretation modes according to the characteristics of specific events and create more opportunities for shared work experiences with team members. This study aimed to find out how to realize the positive effects of inconsistent mental models by the moderating roles of interpretation, providing valuable research for workplace deviant cognition.

## Data availability statement

The original contributions presented in the study are included in the article/Supplementary material, further inquiries can be directed to the corresponding author.

## Ethics statement

The participants in this study were selected with approval from Qingdao beer, Shuanghui Food, and Baixiang Food. The participants provided their written informed consent to participate in the study.

## Author contributions

YZ and XH conceptualized the paper's framework and models. YZ primarily contributed to the investigation and drafted the manuscript, and provided final approval for the manuscript. FR and YG contributed to the data analysis and reviewed the manuscript. All authors contributed to the article and approved the submitted version.
