# Supplementary material for: Do inconsistent mental models impact performance? Moderating effects of managerial interpretation and practice sets
Source: Front Psychol. 2023 Apr 4;14:1110785. doi: 10.3389/fpsyg.2023.1110785 (PMC10112519; doi:10.3389/fpsyg.2023.1110785)
Supplement: Supplementary file 1 [file Data_Sheet_1.docx]

**Appendix A:** Coping Approaches Questionnaire (events 1 to 3)

| **Event 1** (the sales problem in firm QP)  *Given that sales of new products in your department were poor in the past month, please rate the importance of the following coping approaches for addressing the problem, on a scale from 1 (most important) to 9 (least important).* | |
| --- | --- |
| **Coping Approaches** | **Rank (1–9)** |
| 1. Give the agent more benefits. |  |
| 2. Inform the agent about the higher market status of new products and emphasize the opportunities for increasing profits and further development. |  |
| 3. Strengthen your personal relationship with the agent. |  |
| 4. Increase benefits to terminal merchants (restaurants or supermarkets). |  |
| 5. Explain to terminal merchants that new products have a higher market status and greater future benefits. |  |
| 6. Explain to terminal merchants the merits of new products and how they differ from other products. |  |
| 7. Strengthen terminal merchants. |  |
| 8. Survey and change the prices of new products. |  |
| 9. Display prominent advertising in terminal merchants. |  |

| **Event 2** (the sales problem in firm SH)  *In the event of a customer complaint about a product’s quality, please rank the importance of the following coping approaches for addressing the problem, on a scale from 1 (most important) to 6 (least important).* | |
| --- | --- |
| **Coping Approaches** | **Rank (1–6)** |
| 1. Connect with and appease the complainant in a timely manner. |  |
| 2. Check the actual quality of the product. |  |
| 3. Identify the complainant’s desired solution through appropriate communication and negotiation skills. |  |
| 4. Offer an incentive such as a new product or financial compensation. |  |
| 5. Sign an agreement forbidding complainants from broadcasting their complaints through the media. |  |
| 6. Provide other complaint channels for the complainant. |  |

| **Event 3** (the marketing problem in firm BX)  *Given queries about the quality of the brand in the market, please rank the following coping approaches for rebuilding customers’ market belief in the brand on a scale from 1 (most important) to 6 (least important).* | |
| --- | --- |
| **Coping Approaches** | **Rank (1–6)** |
| 1. Survey customers about the brand and its quality. |  |
| 2. Investigate the source of the query. |  |
| 3. Trace the reason for the query. |  |
| 4. Investigate thoroughly the problems in internal quality control. |  |
| 5. Examine the marketing channel of the brand. |  |
| 6. Reflect on the brand positioning and strategy. |  |

**Appendix B:** Teamwork Cognition Questionnaire

| *When other team members aim to enhance their sales, how do you think the following actions affect your own performance? (greatly hinders (-2), hinders a little (-1), no influence (0), helps a little (1), helps a lot (2))* | |
| --- | --- |
| **Actions** | **Influence (−2–2)** |
| 1. The other member offers a lower price. |  |
| 2. The other member has a better relationship with the agent. |  |
| 3. The other member keeps his sales experience secret. |  |
| 4. The other member produces more marketing promotion activities. |  |
